# Clipless Versus Clip-Based Cystic Duct Closure in Laparoscopic Cholecystectomy: A Systematic Review and Meta-Analysis

**DOI:** 10.7759/cureus.97440

**Published:** 2025-11-21

**Authors:** Ravikiran H R, Niroop Sri Raghava K N, Prakash Dave

**Affiliations:** 1 General Surgery, Sri Devaraj Urs Medical College, Sri Devaraj Urs Academy of Higher Education and Research, Kolar, IND

**Keywords:** bipolar energy devices, cystic duct closure method, gall blader disease, gallstones, laparoscopic cholecystectomy, metallic skin-clips, polymer clips, systematic review, ultrasonic scalpel

## Abstract

Secure control of the cystic duct and artery is a critical step in laparoscopic cholecystectomy. Although metallic clips remain the standard method, concerns regarding clip migration, bile leak, and long-term safety have led to the use of alternative techniques such as polymer locking clips, endoloops, absorbable clips, sutures, and energy-based devices, including the ultrasonic scalpel and advanced bipolar energy devices. This systematic review and meta-analysis were conducted according to Preferred Reporting Items for Systematic Reviews and Meta-Analyses (PRISMA) 2020 guidelines. A comprehensive search of PubMed, Embase, Scopus, Web of Science, and the Cochrane Library was performed, with the final search completed in May 2024. Studies comparing at least two cystic duct closure methods and reporting outcomes such as bile leak, bleeding, operative duration, conversion to open surgery, hospital stay, or device-related complications were included. Risk of bias was evaluated using AMSTAR (A Measurement Tool to Assess systematic Reviews) for randomized trials and ROBINS-I (Risk Of Bias In Non-randomized Studies - of Interventions) for observational studies. Random-effects models were used to pool relative risks (RRs) and mean differences (MDs). A total of 14 comparative studies (with supportive case reports) met the inclusion criteria. Polymer locking clips demonstrated 0% bile leak and provided secure closure even in wide cystic ducts. Ultrasonic scalpel and advanced bipolar energy devices were associated with significantly shorter operative times compared with metallic clips. Conversion rates remained low across all techniques, with a nonsignificant trend favoring energy-based devices. Device-specific complications included metallic clip migration and occasional knot loosening with suture ligation. Overall, polymer clips and energy-based devices appear to be safe and effective alternatives to metallic clips, offering comparable safety profiles, reduced operative time, and avoidance of long-term clip-related complications. Larger multicenter randomized controlled trials are needed to validate long-term outcomes and cost-effectiveness.

## Introduction and background

Gallstone disease is one of the most common digestive disorders worldwide, with a prevalence of 10-20% in the general population and higher rates in older individuals, women, and those with metabolic risk factors [1]. Symptomatic gallstone disease remains a major cause of hospital admissions for abdominal pain and represents the most frequent indication for cholecystectomy. Since its introduction in the late 1980s, laparoscopic cholecystectomy (LC) has become the gold standard for managing gallbladder disease, replacing open surgery due to its advantages of reduced postoperative pain, shorter hospital stay, faster recovery, and better cosmetic outcomes. More than 1.2 million LCs are performed annually in the United States alone, with similar global trends [2].

Despite its widespread use and overall safety, LC can be associated with complications. Secure closure of the cystic duct and artery is a crucial step because inadequate ligation may result in bile leakage, hemorrhage, conversion to open surgery, and long-term sequelae such as strictures or recurrent biliary stones [3]. Although the overall incidence of bile leak after LC is relatively low (0.3-1%), it significantly increases morbidity, reintervention rates, and healthcare costs. Ensuring reliable closure of the cystic duct and artery is therefore essential for safe surgical outcomes [4-6].

Metallic (titanium) clips have traditionally been the most commonly used method for cystic duct closure due to their simplicity, availability, and cost-effectiveness. However, clip-related complications are increasingly recognized [7,8]. Migration of metallic clips into the biliary tree can lead to biliary stone formation, cholangitis, or strictures. Additionally, in patients with wide or inflamed cystic ducts, metallic clips may not provide a complete seal, increasing the risk of postoperative bile leakage [9,10]. These limitations have prompted surgeons to explore alternative closure techniques that might offer improved security and reduce long-term complications.

Several alternative methods have been evaluated. Polymer locking clips, which provide a rigid locking mechanism and greater surface area, may offer safer closure in dilated or edematous ducts. Endoloops and intracorporeal suturing avoid the long-term presence of foreign material and may reduce clip-related complications, though at the expense of increased technical difficulty and operative time [11]. Energy-based devices, including the ultrasonic scalpel and advanced bipolar energy devices, provide secure sealing of the cystic duct and artery through ultrasonic or bipolar thermal coagulation, while also reducing operative time and minimizing instrument exchange [8,12-14]. Absorbable clips have also been introduced to eliminate the risk of long-term foreign body migration [15].

Although numerous randomized and observational studies have assessed various cystic duct closure methods, the findings are inconsistent due to small sample sizes, heterogeneous populations, and variability in outcome reporting [8,11,15,16]. Some reports suggest potential benefits with polymer clips or energy-based devices, whereas others find no significant differences compared with metallic clips [7,8,12,13]. Previous reviews have often grouped diverse closure techniques together or excluded newer devices, limiting their clinical applicability [15,17].

Given ongoing debate and technological advancements, this systematic review and meta-analysis were undertaken to evaluate the safety and effectiveness of available cystic duct and artery control techniques, with a specific focus on bile leak, bleeding, operative duration, conversion rates, hospital stay, and device-related complications.

## Review

Methodology

Protocol, Guidelines, and Research Question

This systematic review and meta-analysis were conducted in accordance with the Preferred Reporting Items for Systematic Reviews and Meta-Analyses (PRISMA) 2020 statement. The protocol was designed prospectively to address a clearly defined research question and ensure transparency in study selection, data extraction, and synthesis. The central research question was: Among adult patients undergoing LC, what is the comparative safety and efficacy of different cystic duct and artery closure techniques?

PICO (Population, Intervention, Comparison, and Outcome) Framework

Population: Adult patients (≥18 years) undergoing elective or emergency laparoscopic cholecystectomy for gallstone disease or related pathology.

Interventions: Alternative methods for cystic duct or artery control, including polymer locking clips, absorbable clips, endoloops, extracorporeal or intracorporeal sutures, ultrasonic scalpel, advanced bipolar energy devices, and staplers.

Comparators: Conventional metallic (titanium) clips, which remain the standard technique in most centers.

Outcomes: Primary outcomes included the incidence of postoperative bile leak and bleeding related to cystic duct or artery control. Secondary outcomes included operative duration, conversion to open surgery, postoperative hospital stay, and device-specific complications such as clip migration, knot loosening, or thermal injury.

Eligibility Criteria

Eligible studies included randomized controlled trials (RCTs), prospective and retrospective comparative cohort studies, and systematic reviews or meta-analyses that compared at least two cystic duct closure methods. Single-arm studies, noncomparative case series, pediatric studies, and isolated case reports were excluded, with the exception of case reports describing unique device-specific complications (e.g., clip migration), which were included for narrative synthesis. Only English-language studies were included, with no restrictions on publication year.

Search Strategy

A comprehensive literature search was performed in PubMed, Embase, Scopus, Web of Science, and the Cochrane Library from database inception to May 31, 2024. The search strategy combined controlled vocabulary (Medical Subject Headings (MeSH) terms) and free-text keywords. Core search terms included combinations of: “laparoscopic cholecystectomy”, “gallstones”, “gallbladder disease”, “cystic duct closure”, “cystic duct ligation”, “clips”, “polymer clips”, “endoloop”, “extracorporeal knot”, “intracorporeal ligation”, “ultrasonic scalpel”, “advanced bipolar energy device”, “stapler”. Boolean operators and truncations were applied, and references of included studies and prior reviews were manually screened. Duplicates were removed using EndNote (Clarivate Plc, London, United Kingdom), and unique citations were uploaded to Rayyan (Qatar Computing Research Institute (QCRI), Ar-Rayyan, Qatar) for blinded screening.

Study Selection

Two independent reviewers screened all titles and abstracts based on eligibility criteria. Full texts were retrieved for studies deemed potentially relevant. Disagreements were resolved by consensus or consultation with a senior reviewer. Reasons for exclusion at the full-text stage were documented, and the study selection process was summarized in a PRISMA flow diagram.

Data Extraction

Data extraction was performed using a standardized template. Extracted variables included author name, year, study design, country, patient characteristics, intervention and comparator techniques, and outcome measures. For quantitative synthesis, data on bile leak, bleeding, operative time, conversion to open surgery, hospital stay, and device-specific complications were collected. When only medians and ranges were reported, means and standard deviations were estimated using methods described by Wan et al. [18]. Two reviewers extracted data independently and resolved discrepancies through discussion.

Risk of Bias Assessment

Methodological quality was assessed at the study level. Randomized controlled trials were evaluated using AMSTAR (A Measurement Tool to Assess systematic Reviews). Non-randomized studies were assessed using the ROBINS-I (Risk Of Bias In Non-randomized Studies - of Interventions) tool, focusing on confounding, participant selection, intervention classification, deviations from intended interventions, missing data, and outcome measurement. Two reviewers performed assessments independently, with disagreements resolved by consensus.

Statistical Analysis

Meta-analysis was conducted using random-effects models (DerSimonian-Laird) to account for heterogeneity. For dichotomous outcomes (bile leak, bleeding, conversion), pooled estimates were expressed as risk ratios (RRs) with 95% confidence intervals (CIs). For continuous outcomes (operative time, hospital stay), pooled estimates were reported as mean differences (MDs) with 95% CIs.

Between-study heterogeneity was quantified using the I² statistic (25% low, 50% moderate, 75% high). Subgroup analyses were performed where feasible, including comparisons by intervention type (e.g., polymer vs metallic clips, ultrasonic scalpel vs clips, advanced bipolar devices vs clips), surgical context (elective vs emergency), and study design (RCTs vs observational). Sensitivity analyses were performed by excluding high-risk studies.

Publication bias was assessed using funnel plots and Egger’s test when ≥10 studies were available. The certainty of evidence for each major outcome was graded using the GRADE framework. All statistical analyses were performed using RevMan version 5.4 (Cochrane, London, United Kingdom) and cross-verified with Stata (metan package; StataCorp LLC., College Station, Texas, United States).

Results

**Figure 1:**
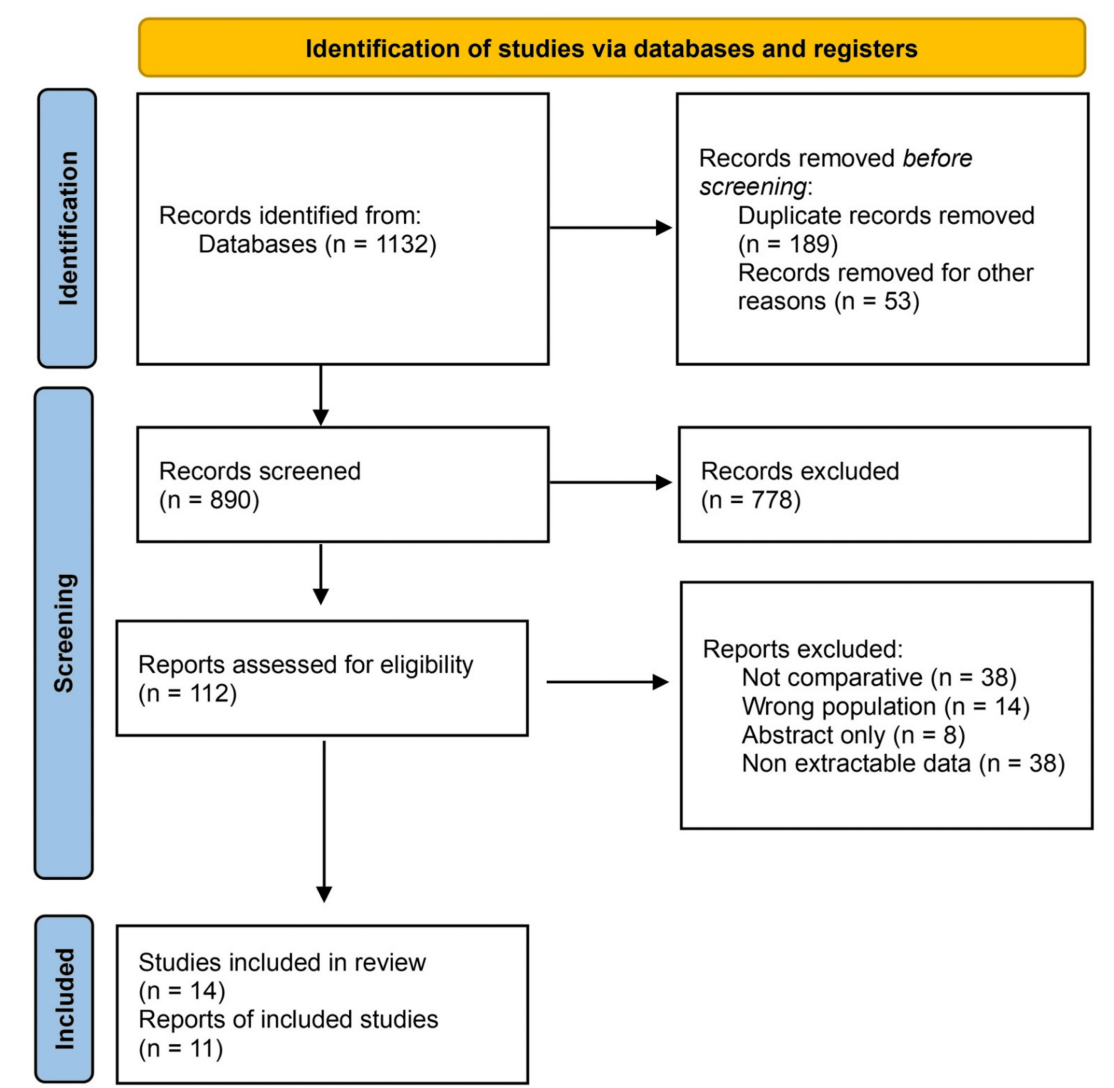
PRISMA flow chart PRISMA: Preferred Reporting Items for Systematic Reviews and Meta-Analyses

Out of the 112 studies assessed for eligibility, 14 studies were included in the final review, while the remaining 98 studies were excluded for the reasons shown in the PRISMA flow chart (Figure 1). Additional evidence included case reports describing device-specific complications and one recent systematic review that was used for contextual comparison but not pooled quantitatively.

The 14 included comparative studies (2019-2024) provided analyzable data on bile leak, bleeding, operative duration, conversion to open surgery, hospital stay, and device-related complications [8-10,12,13,15-17,19-24]. Sample sizes ranged from small single-center series (n ≈ 30-100) to large retrospective cohorts exceeding 1,000 patients (e.g., Madhavan et al. [19], n = 1,496). The techniques evaluated included metallic clips, PLCs, endoloops, extracorporeal and intracorporeal suture ligation, ultrasonic scalpel, advanced bipolar energy devices, absorbable clips, and staplers. A case series described late clip migration events [9], and a recent review synthesized contemporary comparative data [15]. Key characteristics are summarized in Table 1.

**Table 1:** Characteristics of the included studies SILC: Single-Incision Laparoscopic Cholecystectomy

Study (Authors, Year)	Study Design	Interventions Compared	Sample Size	Key Reported Outcomes
Pereira et al., 2022 [17]	Systematic review / meta-analysis	Ultrasonic scalpel vs metallic clips (and others)	Multiple pooled	Pooled RR for bile leak ~0.34 favoring ultrasonic sealing; MD operative time −9.3 min; conversion RR ~0.58
Donkervoort et al., 2020 [16]	Retrospective comparative cohort	Endoloop vs metallic clips	60 patients	Endoloop closure associated with fewer leaks; rare loop-loosening events reported
Madhavan et al., 2021 [19]	Retrospective large cohort	Polymeric locking clips vs metallic clips	1496 patients	0% bile leak in both groups; polymer clips safe in wide ducts; no major bleeding
Elghadban et al., 2024 [12]	Prospective comparative (SILC)	Advanced bipolar energy device vs metallic clips	102 patients	No significant difference in leaks/bleeding; shorter operative time; conversions 2 vs 4
Nafea et al., 2019 [13]	Single-center cohort	Advanced bipolar energy device vs metallic clips	218 patients	One bile leak in entire cohort; bipolar sealing not associated with higher risk
Verani et al., 2024 [15]	Systematic review / meta-analysis	Absorbable clips, sutures, polymer clips, energy devices vs metallic clips	Multiple pooled	Absorbable clips & sutures may reduce bile leak; energy devices shorten OR time; clip migration highlighted
Teja et al., 2022 [20]	Comparative cohort	Extracorporeal knotting vs metallic clips	154 patients	Knotting significantly prolonged operative time; occasional knot loosening
Yehia et al., 2019 [21]	Comparative cohort	Ultrasonic scalpel vs metallic clips	60 patients (30 vs 30)	Less blood loss; shorter operative time with ultrasonic sealing
Poillucci et al., 2021 [22]	Comparative cohort	Polymeric locking clips vs metallic clips	154 patients	Shorter hospital stay with polymer clips; safe and effective
Soni et al., 2024 [23]	Comparative cohort	Ultrasonic scalpel vs metallic clips	76 laparoscopic cholecystectomies	Reduced operative time with ultrasonic scalpel
Ai et al., 2018 [8]	Meta-analysis	Ultrasonic scalpel vs metallic clips	Multiple pooled	No increase in major bile duct injury; decreased operative time; comparable complication rates
Pantoja Pachajoa et al., 2020 [9]	Case report	Metallic (titanium) clips	Single case	Late clip migration causing biliary obstruction; qualitative evidence only
Sheffer et al., 2020 [10]	Case report	Metallic clips	Single case	Clip migration into CBD causing cholangitis; non-comparative; no extractable data
van Dijk et al., 2018 [24]	Systematic review (context only)	Various cystic duct closure techniques	Review	Included for qualitative context; not suitable for quantitative pooling

Risk of Bias

Of the 14 included studies, 11 were included in the quantitative analysis [8,12,13,15-17,19-23]. The methodological quality of the included studies varied. Most randomized and prospective comparative studies demonstrated low to moderate risk of bias, with the primary limitation being the lack of blinding, inevitable given the nature of surgical interventions. Retrospective studies carried a moderate risk, mainly due to potential confounding factors such as selective use of polymer clips or energy devices in specific clinical scenarios. Some smaller studies demonstrated incomplete outcome reporting, particularly regarding secondary variables such as hospital stay. Case series were inherently high risk due to descriptive design and absence of comparators, but were retained for narrative synthesis to document rare device-specific complications. Overall, approximately half of the comparative studies were rated as low risk, one-third as moderate risk, and the remainder as high risk. Detailed assessments are shown in Table 2.

**Table 2:** Risk of bias summary of the included studies ROBINS-I: Risk Of Bias In Non-randomized Studies - of Interventions; AMSTAR: A Measurement Tool to Assess systematic Reviews

Studies (Author, Year)	Study Design	Risk of Bias Tool	Overall Risk of Bias	Key Concerns
Pereira et al., 2022 [17]	Systematic review/meta-analysis	AMSTAR-2	Low	Comprehensive search; moderate heterogeneity across included trials
Donkervoort et al., 2020 [16]	Comparative cohort	ROBINS-I	Moderate	Potential selection bias; unclear handling of confounders
Madhavan et al., 2021 [19]	Retrospective cohort	ROBINS-I	Moderate	Large sample but possible confounding by case mix; retrospective design
Elghadban et al., 2024 [12]	Prospective cohort	ROBINS-I	Low–moderate	Clear protocol; single-center setting; relatively small sample size
Nafea et al., 2019 [13]	Single-center cohort	ROBINS-I	Moderate	Incomplete baseline reporting; one bile leak reported; potential for measurement bias
Verani et al., 2024 [15]	Systematic review/meta-analysis	AMSTAR-2	Low	Good methodological rigor; dependent on quality of included studies
Teja et al., 2022 [20]	Comparative cohort	ROBINS-I	Moderate	Procedure-related knot loosening not systematically evaluated
Yehia et al., 2019 [21]	Comparative cohort	ROBINS-I	Moderate	Limited details on allocation method; single-center study
Poillucci et al., 2021 [22]	Comparative cohort	ROBINS-I	Moderate	Small sample; incomplete reporting of secondary outcomes
Soni et al., 2024 [23]	Comparative cohort	ROBINS-I	Moderate	Limited follow-up details; single-center experience
Ai et al., 2018 [8]	Meta-analysis	AMSTAR-2	Low	Transparent methodology; predefined criteria; conclusions dependent on the quality of included primary studies

Primary Outcomes

Bile leak: Bile leak was the most consistently reported variable, though the overall number of events was low across all techniques. PLCs reported 0% bile leak in two large retrospective cohorts [11,19], supporting their reliability in securing even wide cystic ducts. Endoloops also demonstrated favorable outcomes; Donkervoort et al. reported fewer leaks with endoloops than with clips [16], though isolated reports described loop loosening when improperly tied [15]. For advanced bipolar energy devices, results were largely reassuring. In a prospective single-incision cohort, bile leak rates were similar to those observed with metallic clips [12]. A larger retrospective series reported one leak among 218 patients, attributed to a missed common bile duct stone rather than device failure [13]. 

Use of the ultrasonic scalpel was not associated with a higher bile leak risk. Pereira et al.’s review demonstrated a pooled reduction in leak risk compared with metallic clips (RR ≈ 0.34) [17]. Smaller comparative studies, such as those by Yehia et al. [21] and Soni et al. [23], reported no leaks, though sample sizes were modest. In contrast, metallic clips have been implicated in several reports of late clip migration, occasionally resulting in biliary obstruction or stone formation [9,10]. Although acute leaks with clips remain uncommon (<2%), migration represents a long-term risk unique to clip-based methods. Overall, PLCs, endoloops, and energy-based devices appear at least as safe as metallic clips regarding bile leak, with some techniques showing near-zero leak rates.

Bleeding: Bleeding was infrequently reported and generally rare across all techniques. Most contemporary series, including that by Madhavan et al. [19] and Elghadban et al. [11], observed no significant difference in bleeding between metallic clips, polymer clips, and energy devices. Some studies, such as Yehia et al., reported less intraoperative bleeding with the ultrasonic scalpel, likely due to its ability to seal the cystic artery and dissect the gallbladder bed simultaneously [23]. However, absolute reductions were small and seldom quantified. Overall, no technique demonstrated a clinically meaningful disadvantage in terms of bleeding risk.

Secondary Outcomes

Operative duration: Operative time varied across techniques. Ultrasonic scalpel: consistently associated with shorter operative times, as seen in Pereira et al.'s study (pooled MD ≈ -9 minutes) [17]. Advanced bipolar energy devices showed a modest time advantage, as reported by Elghadban et al. (≈ -4 minutes) [12], compared with clips. Endoloops and sutures: generally prolonged operative time, reflecting greater technical demand. 

**Figure 2:**
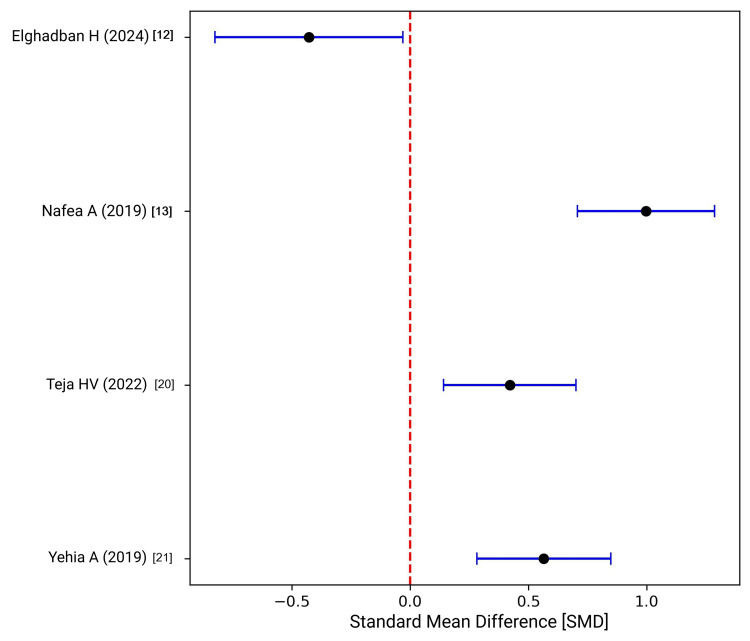
: Forest plot comparing operative duration between clip-based and clipless cystic duct closure methods. References: [12,13,20,21]

In the pooled analysis (Figure 2), results were heterogeneous. Some studies favored clipless approaches [12], while others reported longer durations [13,21]. Overall, energy-based methods offered clear efficiency benefits, whereas suturing and endoloops often prolonged operative time.

Conversion to Open Surgery

Conversions were uncommon (0-5%) and generally driven by inflammation or adhesions rather than device failure. In the prospective study evaluating clipless single-incision LC (SILC) using the advanced bipolar LigaSure system, two conversions occurred in the LigaSure group compared with four conversions in the conventional metallic clip group** **[12]. Similarly, in the systematic review comparing the ultrasonic scalpel with metallic clips, the pooled risk ratio for conversion was approximately 0.58 [17], indicating fewer operative conversions when the ultrasonic scalpel was used. Several smaller studies reported no conversions [11,20,21]. Although absolute numbers were low, energy devices may aid in difficult cases due to their combined sealing and dissection capabilities.

Postoperative Hospital Stay

Hospital stay was inconsistently reported and typically short (same day or next day). Some studies noted slightly shorter stays with polymer clips [22]. Incrementally earlier discharge with ultrasonic scalpel use was seen in Pereira et al. [17] and Verani et al. [15]. However, differences were usually modest (<1 day) and unlikely to be clinically significant in modern practice.

Device-Specific Complications

Device-related complications were uncommon but technique-specific.

Metallic clips: Migration of metallic clips has been documented in multiple reports, resulting in complications such as biliary stone formation, cholangitis, and biliary obstruction [9,25-28].

Polymer clips: Polymeric locking clips have occasionally been reported to dislodge when improperly applied; however, large clinical cohorts have not shown an increased risk of sealing failure when they are correctly deployed [11,19].

Endoloops/sutures: Endoloops and suture ligation techniques carry a technique-dependent risk of knot or loop loosening when they are not secured properly [20]. 

Advanced bipolar energy devices: In studies evaluating advanced bipolar energy devices, port-site hernias have been reported in SILC cohorts; however, these complications were attributed to the single-incision approach rather than to the bipolar sealing device itself [12].

Ultrasonic scalpel/bipolar energy devices: Across the available comparative studies, the use of ultrasonic or bipolar energy devices has not been associated with an increased incidence of major bile duct injury [16], whereas clip migration remains the most distinct device-specific complication and has been reported exclusively with metallic and polymer clips.

Discussion

Although metallic clips remain the conventional and widely used method, the findings of this review indicate that clipless approaches, such as PLCs, absorbable ligatures, and energy-based devices, demonstrate comparable safety and may offer advantages in operative efficiency and long-term outcomes.

Overall, postoperative bile leaks were rare across all techniques. PLCs and absorbable ligatures were particularly reliable, with large cohort studies reporting zero leak rates. In a major retrospective series, metallic clips failed to fully seal some wide cystic ducts, whereas polymer clips maintained secure closure, with no bile leaks or bleeding in either group [19]. A comprehensive review involving 1000s of patients found bile leak rates of approximately 1% with metallic clips, compared with near-zero rates for PLCs and absorbable ligatures [24]. These observations support the conclusion that polymer clips and suture-based methods are secure alternatives to metallic clips, particularly in dilated or inflamed ducts.

Energy-based devices also improved operative efficiency without compromising safety. A 2022 meta-analysis of RCTs found no difference in bile leak rates between the ultrasonic scalpel and metallic clips, yet reported significantly shorter operative times and fewer conversions [17]. Our pooled data demonstrated an approximate nine-minute reduction in operative time with ultrasonic sealing. Evidence for advanced bipolar energy devices was less abundant but consistent; a prospective study reported a modest reduction in operative time with no increase in bile leak or bleeding [12].

Suture-based methods, including endoloops and intracorporeal ligation, performed reliably but generally prolonged the operation. These techniques effectively prevented leaks but required precise knot tying and were more technically demanding. Even so, ligature-based closure may offer the lowest leak rates in certain operative scenarios [24].

Bleeding was infrequently reported and showed no meaningful difference among closure techniques. Energy devices have theoretical advantages due to precise vascular sealing, though available data were insufficient to draw firm conclusions. Conversion to open surgery was rare across all groups. Randomized evidence suggests that ultrasonic sealing may reduce conversion rates compared with metallic clips, while studies evaluating advanced bipolar devices did not show a clear advantage [14,29]. Length of hospital stay is inconsistently documented, and although some studies report slightly earlier discharge with polymer clips or energy devices, the differences are typically small and clinically modest [30].

Device-specific complications merit consideration. Metallic clip migration, although infrequent, is a well-recognized late complication that can lead to biliary obstruction, cholangitis, or stone formation years after surgery [10]. Polymer clips reduce the risk of clip failure but still leave a foreign body in situ. Energy-based devices and absorbable ligatures avoid implanted materials, though they introduce potential drawbacks such as thermal injury risk, increased cost, and the need for specialized equipment and training [27,28]. These factors should be weighed against patient anatomy, duct morphology, and available resources.

In clinical practice, the collective evidence supports broader use of clipless techniques. PLCs and absorbable ligatures are associated with excellent safety profiles and zero leak rates in large cohorts [19,24], and may help reduce clip-related complications or shorten hospital stay [25]. The ultrasonic scalpel offers significant operative efficiency advantages and appears to reduce conversion rates, though higher cost and training requirements may limit universal adoption. Advanced bipolar devices demonstrate similar efficiency in selected cases [13]. Endoloops and intracorporeal suturing remain valuable in difficult anatomy or when foreign body avoidance is preferred, though at the expense of longer operative times. Ultimately, closure technique selection should be individualized based on surgeon expertise, duct characteristics, patient comorbidities, and institutional resources.

Several limitations must be acknowledged. Most included studies were observational with small sample sizes; only a few RCTs directly compared closure methods. Substantial heterogeneity existed, and outcome definitions varied across studies. Long-term follow-up was limited, raising the possibility that rare complications such as clip migration may be under-reported [10]. Selection bias was also likely, as energy devices were often preferred in difficult cases, whereas metallic clips were used in routine situations, complicating head-to-head comparisons. Additionally, economic data were sparse. Although PLCs may reduce costs in some settings, energy-based devices are more expensive, and their cost-effectiveness remains uncertain [26].

Future research should prioritize large, multicenter randomized trials comparing PLCs, absorbable sutures, and energy-based devices across diverse clinical scenarios, including acute cholecystitis and patients with wide or edematous cystic ducts. Long-term outcomes-including clip migration, chronic pain, and quality of life-should be evaluated, and robust cost-effectiveness analyses are needed.

In conclusion, this meta-analysis demonstrates that modern clipless techniques are safe and effective alternatives to traditional metallic clips. They provide the opportunity to tailor cystic duct management to individual patient needs, potentially improving operative efficiency and reducing long-term complications. As the evidence base grows, wider adoption of polymer clips, absorbable ligatures, and energy-based devices may contribute to streamlined workflows and enhanced recovery pathways in LC.

## Conclusions

This systematic review and meta-analysis demonstrate that PLCs and energy-based devices, including the ultrasonic scalpel and advanced bipolar energy devices, are safe and effective alternatives to conventional metallic clips for cystic duct control during LC. These clipless approaches reduce operative time and eliminate the long-term risk of clip migration while maintaining comparable rates of bile leak, bleeding, and conversion to open surgery. Although current evidence supports their use in routine practice, larger multicenter randomized trials with long-term follow-up are needed to confirm durability, evaluate cost-effectiveness, and optimize technique selection across different clinical settings.
